# The clinical characteristics and gene mutations associated with thyroid hormone resistance syndrome coexisting with pituitary tumors

**DOI:** 10.3389/fendo.2023.1131044

**Published:** 2023-02-10

**Authors:** Junyu Zhao, Lusi Xu, Chunyu Li, Fei Wang, Lin Liao, Jianjun Dong

**Affiliations:** ^1^Department of Endocrinology and Metabology, The First Affiliated Hospital of Shandong First Medical University and Shandong Provincial Qianfoshan Hospital, Shandong Key Laboratory of Rheumatic Disease and Translational Medicine, Shandong Institute of Nephrology, Jinan, China; ^2^Department of Endocrinology and Metabology, Shandong Provincial Qianfoshan Hospital, Shandong University, Jinan, China; ^3^Department of Endocrinology and Metabology, Qilu Hospital of Shandong University, Cheeloo College of Medicine, Shandong University, Jinan, China

**Keywords:** thyroid hormone resistance syndrome, pituitary tumors, *THRβ*, clinical characteristics, gene mutation

## Abstract

**Aims:**

Resistance to thyroid hormone (RTH) and pituitary tumors are both rare diseases, and the differential diagnosis of these two diseases is difficult in some cases. There are also patients who have both conditions, making diagnosis more difficult. To better understand this aspect, we analyzed the clinical characteristics and gene mutations of RTH coexisting with pituitary tumors.

**Methods:**

Database retrieval was conducted in the PubMed, Cochrane Library, and SinoMed databases, and the search contents were case reports or case series of patients with RTH coexisting with pituitary tumors. The demographic, clinical manifestations, and imaging characteristics of pituitary tumors and gene mutations were summarized.

**Results:**

Thirteen articles involving 16 patients with RTH coexistent with pituitary tumors, consisting of 13 female patients, one male patient, and two patients with unknown sex, were included. The patients were 10 to 79 years old and most patients were 41-55 years old (43.75%). The 16 patients were from seven different countries and three continents (Asia, the Americas, and Europe). All the patients showed an abnormal secretion of TSH, and five patients underwent transsphenoidal surgery. Finally, four patients were pathologically confirmed to have TSHoma. A total of 11 different mutations occurred at nine amino acid sequence sites (251, 310, 344, 347, 383, 429, 435, 438, and 453). Two different mutations occurred in both the no. 435 and no. 453 amino acid sequences. Fourteen patients provided their treatment histories, and all had undergone different treatment regimens.

**Conclusions:**

Patients with both RTH and pituitary tumors had multiple clinical manifestations and different thyroid functions, imaging characteristics of pituitary tumors, genetic mutations of *THRβ*, and treatments. However, due to the limited number of cases, the patients were mainly women. Further studies with more cases that focus on the mechanism are still needed.

## Introduction

Resistance to thyroid hormone (RTH) and thyroid-stimulating hormone (TSH)-secreting adenoma (TSHoma) of the pituitary, as the main causes of abnormal secretion of TSH, are clinically rare diseases that are mainly characterized by unregulated thyroid hormone negative feedback, manifested as elevated thyroid hormone with normal or elevated TSH (1–3). RTH is usually caused by mutations in the thyroid hormone receptor-β (*THRβ*) gene and is characterized by a series of clinical manifestations such as tissue insensitivity to the thyroid hormone. Direct sequencing of the *THRβ* gene confirms the diagnosis of RTH in 85% of the cases (4). Since RTH is mainly inherited by autosomal dominant inheritance (it can also be autosomal recessive but is rare), it can be effectively diagnosed by measuring thyroid function in first-degree relatives. An RTH patient without mutations in the beta isoform of the thyroid hormone receptor is also occasionally seen. The diagnosis of RTH is challenging, and it is mainly differentiated from TSHoma. For pituitary tumors, TSHoma cannot be definitively diagnosed without pathological examination. Other clues to help with the diagnosis of TSHoma are visual symptoms caused by pituitary enlargement that presses on the optic chiasma or the abnormal secretion of prolactin or growth hormone from the anterior pituitary. It is interesting that a limited number of reports show that RTH can coexist with pituitary tumors (especially TSHoma) in the same patient (5–17). Sometimes, RTH and TSHoma are difficult to distinguish because some RTH patients may have signs and symptoms of thyrotoxicosis, especially tachycardia, hyperactivity, and hyperreflexes, and many patients have goiters. Conversely, a small number of TSHoma patients have mild symptoms of thyroid toxicity or even none. A correct diagnosis for RTH patients without *THRβ* gene mutations is also difficult, and they are often misdiagnosed. In addition, sporadic pituitary lesions have been reported in up to 24% of patients with RTH (18), thus increasing the complexity of differential diagnosis. Hence, this is the reason why the characteristics of patients with both diseases need to be differentiated. Therefore, we conducted this study to summarize the clinical characteristics and gene mutations of RTH coexistent with pituitary tumors.

## Materials and methods

**Figure 1:**
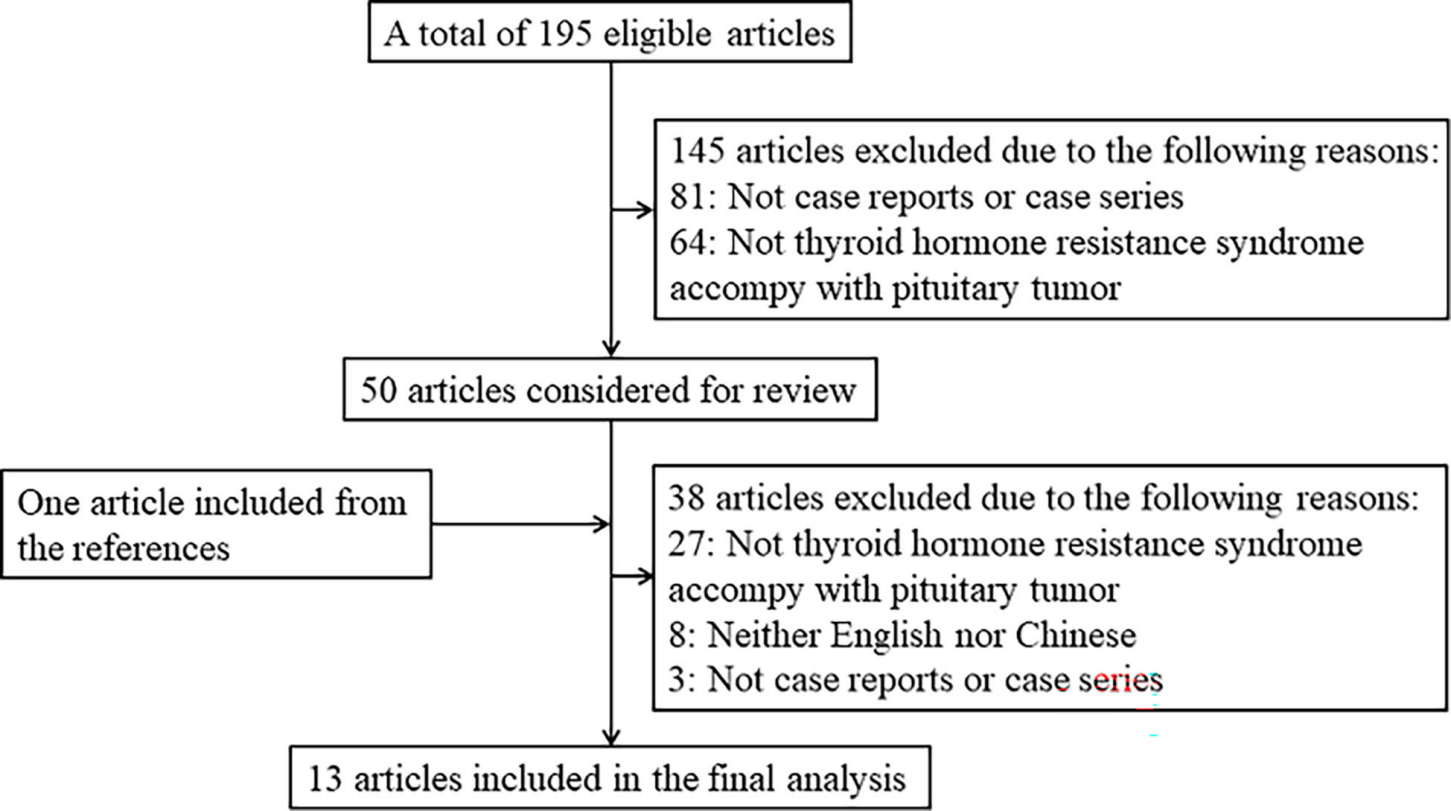
Literature review inclusion process.

We performed a search in PubMed, Cochrane Library, and SinoMed for case reports or case series about patients with thyroid hormone resistance syndrome coexisting with pituitary tumors. We also hand-searched the reference lists of all eligible articles and related previous review articles. The literature search was restricted to published results from the earliest date to 9 December 2022 with the following search terms: ((Thyroid Hormone Resistance Syndrome[MeSH Terms]) OR (resistance to thyroid hormone) OR (RTH) OR (Thyroid hormone resistance syndrome) OR (resistance to thyroid hormone syndrome) OR (pituitary resistance to thyroid hormone) OR (PRTH)) AND ((Pituitary Neoplasms[MeSH Terms]) OR (hypophysoma) OR (pituitary adenoma) OR (pituitary tumor)). Studies that met the following criteria were included in this article: 1) published in English or Chinese language, 2) case reports or case series, 3) subjects were patients with RTH syndrome coexistent with a pituitary tumor, and 4) clinical characteristics and genetic mutations were described. Otherwise, a study was not included in the subsequent analysis. The flowchart shows the literature review inclusion process (**Figure 1**).

The following information was extracted from the eligible articles: country; number of patients; and characteristics of cases including age, sex, size of pituitary tumor, symptoms or diseases, thyroid function (FT3, FT4, and TSH), and molecular biology results (including gene mutations and protein). The demographic, clinical, and genetic mutation information of the cases above was described utilizing simple summary statistics.

## Results

### General data

**Figure 2:**
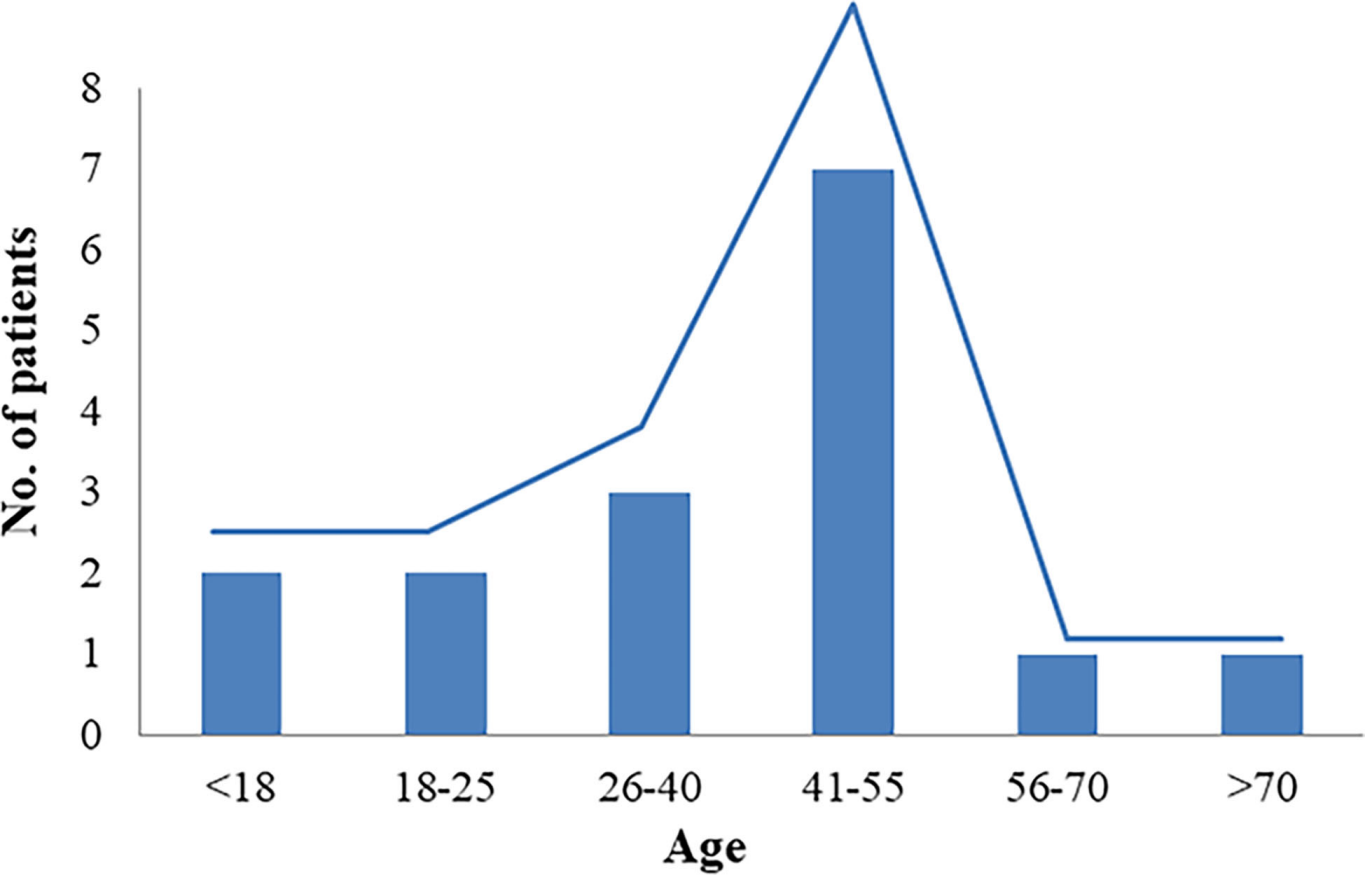
Patients’ age distribution.

**Figure 3:**
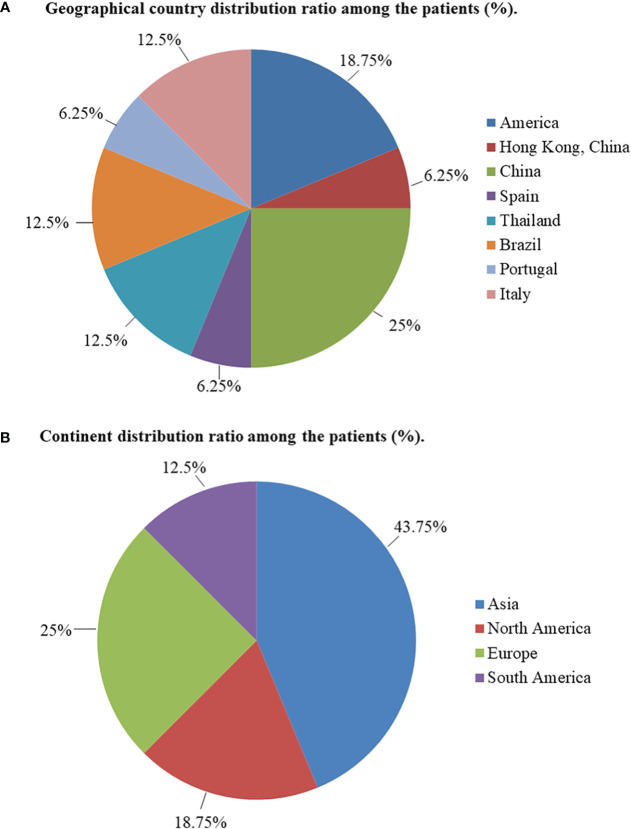
**(A)** Geographical country distribution ratio among the patients (%). **(B)** Continent distribution ratio among the patients (%).

This study included 13 articles (**Figure 1**) that were first published in 2001 and recently published in 2022. In total, the articles involved 16 patients with RTH syndrome coexisting with pituitary tumors, consisting of 13 female patients, 1 male patient, and two patients with unknown sex. The patients were 10 to 79 years old. The age group with the largest number of patients was 41-55 years old, accounting for 43.75% of the total number of patients, followed by the 26-40-year-old group (18.75%). There was no significant difference in the number of patients under 25 years old (four patients) and over 56 years old (two patients) (**Figure 2**). The 16 patients were from seven different countries: five from China (one from Hong Kong, China); three from America; six from Thailand, Brazil, and Italy, each with two patients; and one each from Spain and Portugal (**Figure 3A**). Patients were grouped according to the distribution of the continents, and Asia accounted for the largest proportion of patients (43.75%), followed by the Americas (18.75% in North America and 12.5% in South America). No cases have been reported in Oceania or Africa (**Figure 3B**).

### Clinical manifestations

Clinical symptoms varied among the 16 patients included in the analysis. Three patients did not provide any information about symptoms (6, 17), and another three patients had no clinical symptoms (11, 12). Palpitations were reported in six patients (5, 8–10, 14, 17), two patients had cervical swelling (7, 13), and one patient was suspected to be diagnosed with hypothyroidism because she had several typical symptoms, such as fatigue, weakness, weight gain, memory loss, dry eyes, anxiety, and depression (7). Among these 16 patients, four did not have a physical examination of the thyroid (6, 16, 17). Eight patients had goiter (5, 8–10, 12–15), and three had thyroid nodule(s) (12, 13, 15). Prior to a definitive diagnosis of RTH, most of the patients (9/16) were diagnosed with hyperthyroidism or thyrotoxicosis due to clinical symptoms and elevated thyroid hormone levels (5, 6, 8, 9, 12, 14, 15, 17). All sixteen patients showed abnormal TSH secretion, such as elevated thyroid hormone, but normal or elevated TSH. The thyroid function details (FT3, FT4, and TSH) of the 16 patients are summarized in **Table 1**.

**Table 1:** Summary of thyroid function.

First author, year	FT3	FT4	TSH
Ando S, 2001-1	NA	7.4 ng/dl (0.9-1.6)	10.5 μU/ml (0.43-4.60)
Ando S, 2001-2	NA	1.7 ng/dl (0.9-1.6)	121.6 μU/ml (0.43-4.60)
Safer JD, 2001	606 pg/dl (210-440)	2.6 ng/dl (0.8-2.7)	0.4 mIU/L (0.3-5.0)
Kong AP, 2005	9.65-12.3 pmol/L (3.28-8.2)	20.7 pmol/L (8.5-20.7)	16.7 mIU/L (0.3-4.0)
Teng X, 2015	14.25 pmol/L (2.63-5.7)	28.79 pmol/L (9.01-19.05)	21.11 mIU/L (0.35-4.94)
Ramos-Leví AM, 2016	7.32 pg/ml (2.5-3.9)	2.2 ng/dl (0.8-1.7)	10.5 μU/ml (0.3-5.6)
Sriphrapradang C, 2016	NA	13.9 (6-11.5)	2.8 mU/L (0.4-3.6)
Ramos LS, 2018	NA	2.8 ng/dl (0.6-1.5)	1.5 mIU/L (0.3-4.0)
	NA	2.9 ng/dl (0.6-1.5)	6.7 mIU/L (0.3-4.0)
Yu C, 2018	9.76 pg/ml (4.1-7.9)	26.78 pg/ml (12-22)	5.21 μIU/ml (0.27-4.2)
Carvalho Cunha N, 2019	NA	2.1 pg/dl (0.8-1.9)	9.6 μIU/ml (0.4-4.0)
Jiaqi L, 2019	NA	56.7-70.7 pmol/L (12.0-22.0)	4.85-10.93 mU/L (0.27-4.20)
	NA	32.34-32.09 pmol/L (12.0-22.0)	10.73-26.34 mU/L (0.27-4.20)
Campi I, 2020	8.13 pmol/L (4.2-7.5)	35.3 pmol/L (10-20)	1.3 μIU/ml (0.3-5.1)
	6.9 pmol/L (4.2-7.5)	26.8 pmol/L (10-20)	1.58 μIU/ml (0.3-5.1)
Suntornlohanakul O, 2022	3.14 pg/ml (2-4.4)	2.09 ng/dl (0.7-1.75)	7.27 mIU/L (0.25-4)

FT3, free triiodothyronine; FT4, free thyroxine; TSH, thyroid-stimulating hormone; NA, not available.

### Imaging characteristics of the pituitary tumors

Pituitary MRI examination was abnormal in all 16 included patients. One patient did not report the size of the pituitary tumor (8), four patients were diagnosed with macroadenoma with diameters greater than 10 mm (5, 6, 10, 17), and the remaining 11 patients were diagnosed with microadenoma (7, 9, 11–16). Five patients underwent transsphenoidal surgery (5, 6, 8, 9, 16), and four patients were confirmed to have TSHoma by a pathological examination (5, 6, 9, 16). One patient was diagnosed with a pituitary cyst (16), another was diagnosed with pituitary hyperplasia (17), and the remaining 10 were all diagnosed with pituitary adenoma (7, 8, 10–15) either by a pituitary MRI scan or pathological examination.

### Gene mutations

**Figure 4:**
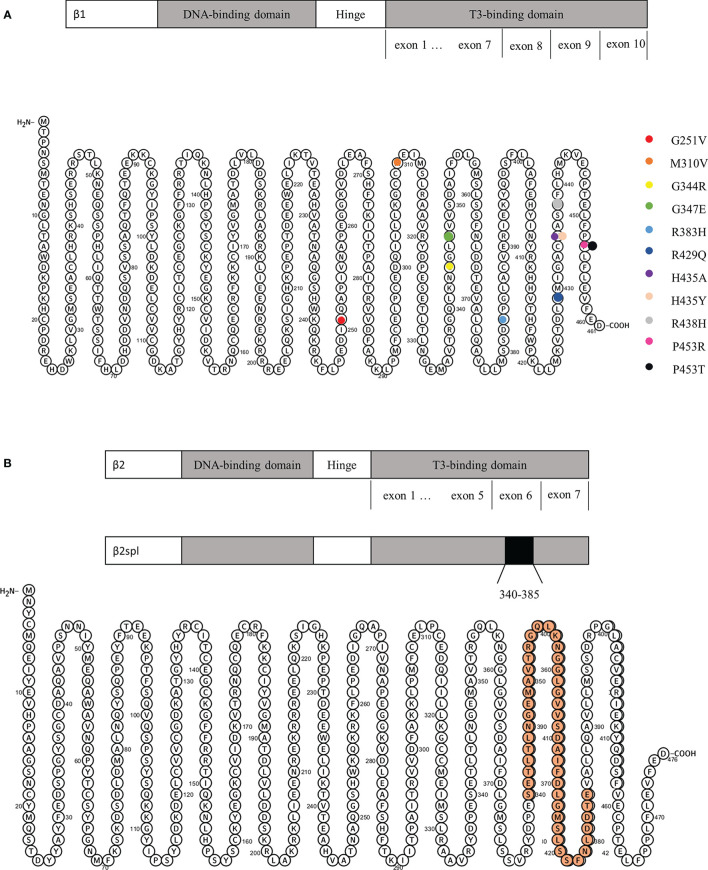
**(A)** Schematic representation of *THRβ1* and its gene mutations. **(B)** Schematic representation of *THRβ2* and its deletion from amino acids 340 to 385.

All 16 patients underwent genetic testing, and 15 of them had abnormalities in the *THRβ* and were diagnosed with RTH. RTH without mutations in the *THRβ* gene (non-TR-RTH) was considered when patients did not have mutations in the beta isoform of the THR but had other characteristics of RTH according to laboratory evaluations, such as the α-subunit, TRH stimulation test, T3 suppression test, and sexual hormone binding globulin. Finally, one patient was diagnosed with non-TR-RTH (12). Ando S et al. (5) reported aberrant alternative splicing of *THRβ2* (135 bp deletion) that caused hormonal dysregulation and hormone resistance (**Figure 4B**). The details of the gene mutations in the 16 patients are shown in **Table 2**. A total of 11 different mutations occurred at nine amino acid sequence sites (251, 310, 344, 347, 383, 429, 435, 438, and 453), and a schematic representation of *THRβ1* is shown in **Figure 4A**. These mutations involve the following gene loci: c.1030G>A, c.1037G>T, c.1040G>A, c.1286G>A, c.1303C>A, c.1433G>A, c.1642C>A, and c.1642C>G. Two different mutations occurred in both the no. 435 and no. 453 amino acid sequences. Three patients had the same point mutation in the no. 429 amino acid sequence causing the replacement of normal arginine with glutamine (8, 15, 16), and two patients had the same point mutation in the *THRβ* gene (c.1642C>A), leading to a missense change of proline 453 to threonine (p.Pro453Thr) (9, 10). No relationship was found between the site and types of genetic mutations and the patients’ country, age, sex, thyroid function, and pituitary tumor characteristics by further analysis.

**Table 2:** Summary of the *THRβ* mutations of RTH patients coexistent with pituitary tumor.

Study	Gender	Gene mutations	Protein
Ando S, 2001-1	F	THRβ2spl has a deletion from amino acids 340 to 385	
Ando S, 2001-2	F	c.1303C>T	p.His435Tyr
Safer JD,2001	F	c.1313G>A	p.Arg438His
Kong AP, 2005	M	c.1286G>A	p.Arg429Gln
Teng X, 2015	F	c.1642C>A	p.Pro453Thr
Ramos-Leví AM, 2016	F	c.1642C>G	p.Pro453Arg
Sriphrapradang C, 2016	F	c.1037G>T	p.Gly251Val
Ramos LS, 2018	F	c.1642C>A	p.Pro453Thr
	F	Without mutations	
Yu C, 2018	F	c.1303C>A	p.His435Ala
Carvalho Cunha N, 2019	F	c.1030G>A	p.Gly344Arg
Jiaqi L, 2019	F	c.1040G>A	p.Gly347Glu
	F	c.1286G>A	p.Arg429Gln
Campi I, 2020	NA	c.1286G>A	p.Arg429Gln
	NA	NA	p.Met310Val
Suntornlohanakul O, 2022	F	c.1433G>A	p.Arg383His

F, female; M, male; NA, not available.

### Treatment

Fourteen patients provided their treatment histories, and all 14 patients had undergone different treatment regimens. Due to TSHoma or suspected TSHoma (finally pathologically confirmed as a pituitary adenoma), five patients underwent transsphenoidal surgery (5, 6, 8, 9, 16). Two patients underwent a thyroidectomy for Graves’ disease or nodular goiter and thyroid adenoma (6, 13). Three patients had received ^131^I therapy for hyperthyroidism or goiter accompanied by increased heart rate and thyroid hormone levels (6, 10, 15). Antithyroid drugs, such as thiamazole (methimazole), were taken by five patients for suspected hyperthyroid (9, 12, 14, 15) or TSHoma (16). Levothyroxine was administered to four patients who were suspected of having hypothyroidism or secondary hypothyroidism after thyroidectomy and ^131^I therapy (7, 10, 13, 15). Only two patients received triiodothyroacetic acid or T3 therapy for RTH (11, 12). Furthermore, most of the patients had abnormal TSH during follow-up.

## Discussion

Abnormal TSH secretion with high levels of FT3 and FT4 and high or normal levels of TSH is due to either a TSHoma or RTH (1–3). Although RTH and TSHoma are both syndromes of abnormal secretion of TSH, their pathogenesis, treatment strategies, and prognosis are completely different. TSHoma, a rare functional pituitary tumor, accounts for less than 1% of all pituitary adenomas (19). Due to the clonal expansion of abnormal cells, the secretion of thyroid hormone is not affected by its negative feedback. Therefore, currently, the first-line treatment is surgical resection. However, the mechanism of TSHoma is still unclear. A hypothesis was proposed by Beck-Peccoz P et al. (1) in 1996 that the downregulation of the thyroid hormone may be one mechanism. In addition, mice with a mutation in the β-isoform of the thyroid hormone receptor spontaneously develop TSHoma (20). This suggests the role of thyroid hormone receptors in pituitary tumors or even predisposes patients to TSHoma (9).

RTH is a disease first reported by Refetoff et al. in 1967 (21), and some of the main causes of RTH include 1) mutation of the thyroid hormone receptor, 2) binding disorder of the thyroid hormone and its receptor, and 3) abnormal post-binding action of the thyroid hormone receptors, etc., leading to the insensitivity of the tissues to the thyroid hormone, thus causing metabolic disorders, thyroid dysfunction, etc. In addition to the brain, testis, and lymph, thyroid hormone receptors are widely expressed in diverse tissues, organs, and cells. The α- and β-isoforms are two subtypes of THR, and mutation or dysfunction is an important cause of RTH. Currently, approximately 80% of RTH is caused by *THRβ* mutations, and approximately 10%–15% of cases are not caused by *THRβ* mutations (18, 22, 23). RTH, as it is often referred to, is mainly a rare autosomal dominant disease characterized by mutation in the β-isoform of the thyroid hormone receptor (2, 3). The *THRβ* gene is located at 3p24.2 and consists of 10 exons. RTH can be divided into two main categories according to the site of RTH and clinical manifestations, that is, generalized RTH and central or pituitary RTH (24). Partial resistance is commonly seen in the clinic, while true complete resistance is rare. However, various organs and tissues have different degrees of resistance to the thyroid hormone, and patients have different compensatory abilities, so there are different clinical manifestations and laboratory characteristics. Clinical manifestations can include hyperthyroidism, normal thyroid function, or hypothyroidism. To date, no efficient therapy has been found to correct the defect in THR function especially that caused by gene mutation. Therefore, symptomatic treatment to relieve the symptoms of hypo- or hyperthyroidism is the main therapy for RTH. For patients with hyperthyroidism, antihyperthyroidism drugs can be used, and thyroid hormone supplementation should be considered. Although the diagnosis was the same, the clinical symptoms, thyroid function, and treatment regimen were all different in the 16 patients in this study. It is important to stress that invasive treatments such as thyroidectomy or radioactive iodine ablation are not needed. Unfortunately, in the few cases we included, some patients underwent unnecessary invasive treatment.

The symptoms and signs of RTH and TSHoma have similar characteristics, such as palpitations, goiter, and similar thyroid function in both diseases. For RTH patients with coexisting pituitary tumors, the signs and symptoms might be worse and diverse, and the abnormalities caused by the high level of prolactin or growth hormone from the anterior pituitary and the visual symptoms caused by pituitary enlargement pressing on the optic chiasma might occur at the same time. Prior to the correct diagnosis of these different diseases, some aggressive treatments may result in serious adverse outcomes, such as inappropriate thyroid ablation in patients with TSHoma or unnecessary pituitary surgery in patients with RTH. These problems lead to the need for further testing, including laboratory tests, dynamic tests, genetic tests, and pituitary imaging. To evaluate patients with abnormal TSH secretion and pituitary imaging abnormalities, combined examination is usually required to distinguish TSHoma from RTH. There have been several previous reports of RTH with non-functional or functional pituitary adenomas (5–17). Both RTH and pituitary tumors are rare diseases, but it is still possible for a patient to have both, although it is highly unlikely. Is there a connection between the two diseases? It is known that up to 15% of people without any diseases may have a small and non-functional pituitary adenoma, and patients with RTH may occasionally have abnormal imaging findings (25). As we mentioned above, thyroid hormone receptors may play a role in the development of pituitary tumors (or TSHoma). In addition, a somatic mutation of *THRβ* was found in a TSH-secreting pituitary adenoma in one of the patients included in our study (5). However, the relationship between RTH and pituitary tumors (especially TSHoma) and whether RTH can promote the occurrence of TSHoma are still unknown due to the limitations of current studies.

Although genetic testing could assist in the diagnosis of RTH, there are still up to 10%–15% of RTH patients without *THRβ* mutations (which we called non-TR-RTH), which may affect the diagnosis of such patients, especially for patients with pituitary tumors, leading to the wrong choice of surgical treatment (4, 26). It has also been described in the literature that 15% of RTH families may have non-TR-RTH patients (27). In this study, we reported a non-TR-RTH patient with pituitary microadenoma, but whether the pituitary microadenoma was a TSHoma was unknown because pituitary pathological examination was not performed.

In this paper, a total of 11 different mutations occurred at nine amino acid sequence sites, but no regular mutations or mutations associated with any particular phenomenon were found. *THRβ1* (widely expressed), *THRβ2* (mainly expressed in the brain, retina, and inner ear), and *THRβ3* (expressed in the kidney, liver, and lung) are the three main T3-binding splicing products of *THRβ* (28, 29). The mutation of *THRβ* has both dominant and negative effects. Point mutations often manifest as autosomal dominant, and a deletion is often autosomal recessive. It was reported that exons 7-10 of *THRβ* mainly encode the T3-ligand-binding region (30), and the mutations reported in patients in this study were mainly concentrated in exons 7-10 encoding the T3-ligand-binding region.

Thyroid disease is more common in women than in men. As an autosomal disorder, the prevalence of RTH is approximately 1/40,000-19,000, and the frequency among the sexes is equal (18, 31). Daly AF et al. (32) reviewed the prevalence studies of clinically relevant pituitary adenomas in the general population that had been published before 2020 and reported that the prevalence of pituitary adenomas differed from 1/865 to 1/1,322. At the same time, it is higher in women than in men (differing from 62.2% to 77.3%). However, it is still unknown whether a sex difference exists in *THRβ* mutation patients. Santos Mata MA et al. (33) summarized a multicentrical case series (a total of 22 RTH patients) and showed that of the 12 patients who underwent THR genetic testing, eight men and three women had *THRβ* mutations and one woman had normal *THRβ* and *THRα*. Interestingly, in our study, except for two patients who did not provide gender information, only one of the other 14 patients was a male patient and the rest (92.86%) were all female patients, which means that the dataset is mostly made of female individuals. In other words, in this dataset, the proportion of female patients with RTH complicated with pituitary tumors was significantly higher than that of male patients. Due to the limitations of the dataset compared to the general incidence of the diseases, the results still need to be supported and verified with more data. At the same time, the mechanism by which sex and RTH coexist with pituitary tumors needs further study.

## Summary

Both RTH and pituitary tumors are rare diseases, and patients diagnosed with both are even rarer. This study shows that patients with both RTH and pituitary tumors have multiple clinical manifestations and different thyroid functions, imaging characteristics of pituitary tumors, genetic mutations of *THRβ*, and treatments. However, due to the limited number of cases, patients were mainly women. Further studies with more cases that focus on the mechanism are still needed.

## Author contributions

JZ and LX: document retrieval, data extraction, data analysis, essay writing, and paper submission. CL and FW: data extraction and data analysis. JD: article innovation. LL: article innovation and paper submission. All authors contributed to the article and approved the submitted version.
